# Supplementary material for: Leveraging the Fragment Molecular Orbital Method to Explore the PLK1 Kinase Binding Site and Polo-Box Domain for Potent Small-Molecule Drug Design
Source: Int J Mol Sci. 2023 Oct 27;24(21):15639. doi: 10.3390/ijms242115639 (PMC10650754; doi:10.3390/ijms242115639)
Supplement: Supplementary file 1 [file ijms-24-15639-s001.zip › ijms-2640953-supplementary.pdf]

Leveraging the Fragment Molecular Orbital Method to Explore the PLK1 Kinase Binding Site and Polo-Box Domain for Potent Small-Molecule Drug Design

Haiyan Jin[2*], Jongwan Kim[1,3*], Onju Lee[2], Hyein Kim[3], and Kyoung Tai No[1,2,3,4.]

[1] Department of Biotechnology, Yonsei University, Seoul, Republic of Korea

[2] The Interdisciplinary Graduate Program in Integrative Biotechnology & Translational Medicine, Yonsei University, Incheon, Republic of Korea

[3] Bioinformatics and Molecular Design Research Center (BMDRC), Incheon, Republic of Korea

[4] Baobab AiBIO Co., Ltd., Incheon 21983, Republic of Korea
[*] Co-first authors

[*] Correspondence: Kyoung Tai No (ktno@yonsei.ac.kr), Jongwan Kim (jwkim@bmdrc.org)

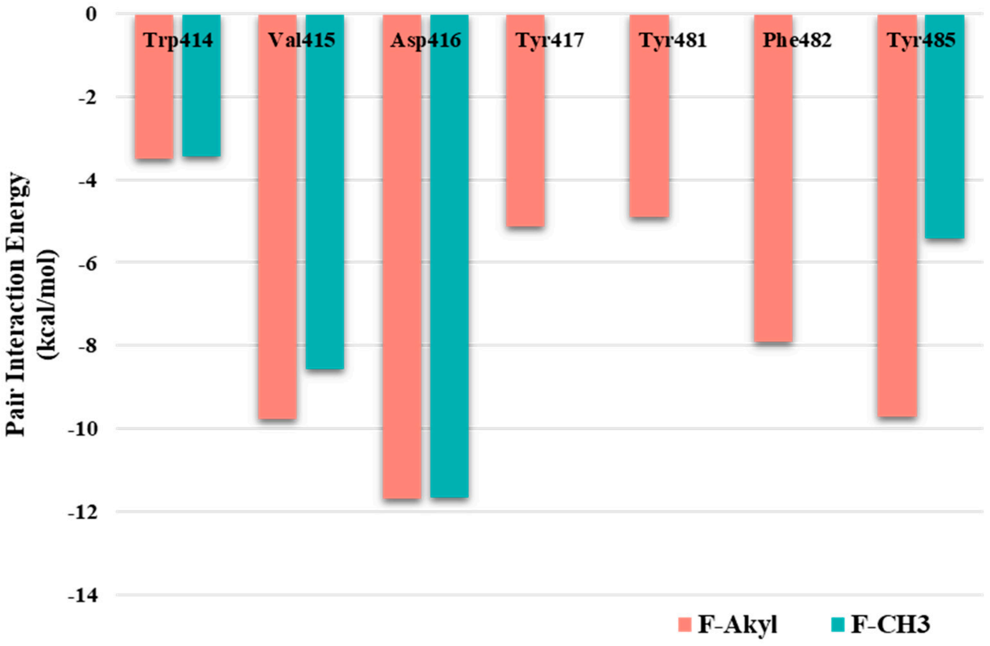

Figure S1. Comparison of F-Akyl with F-CH3 using pair interaction energy (PIE).

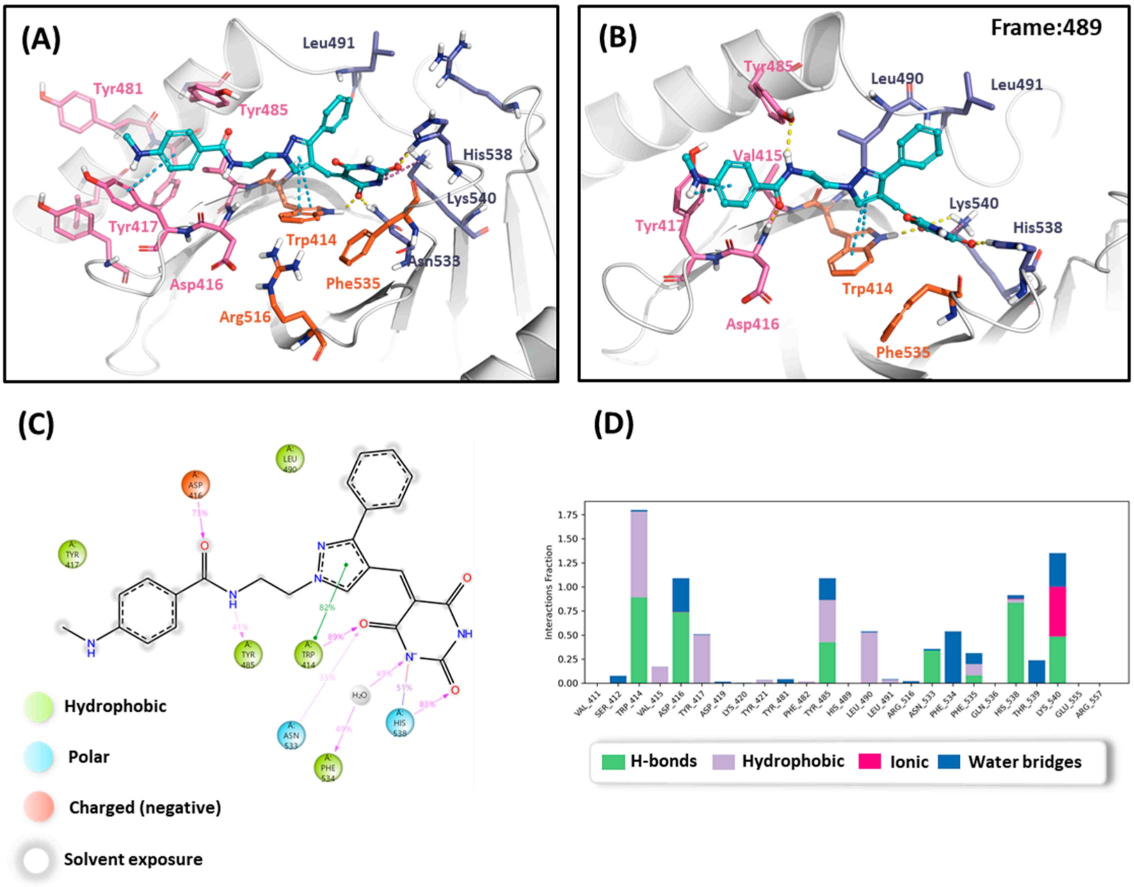

Figure S2. Molecular dynamic (MD) simulation analysis of KBJK557. (A) Docking structure of KBJK557 with the polo-box domain (PBD). (B) Structure of Frame 489 chosen from the MD simulation. (C) Protein-ligand contacts of the two-dimensional (2D) chemical structure and 2D summary reveal that interactions occur more than 30% of the simulation time. (D) Histogram of the protein-ligand interaction fraction.

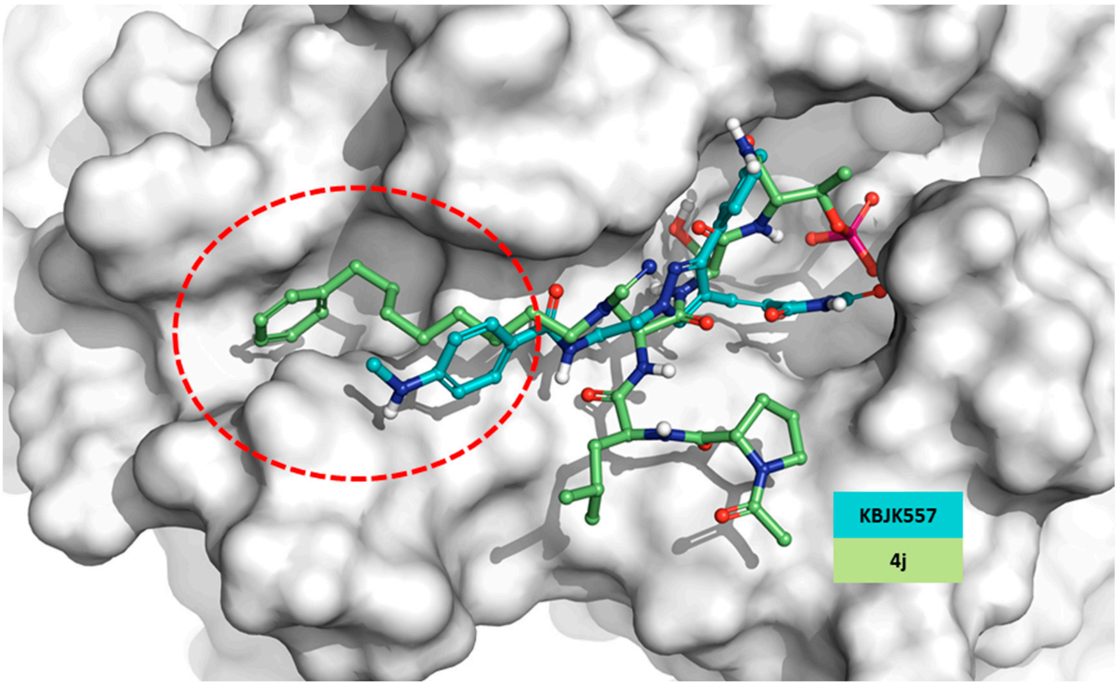

Figure S3. Superposition of 4a and KBJK557. Overlay representation of KBJK557 with 4j, where KBJK557 is light blue, 4j is light green, and the protein is presented with a white surface.

Table S1. PIEDA of ATP and PLK1 complex (PDB ID: 2OU7). All energies are in kcal/mol. The calculation was conducted at the FMO2/DFTB3/PCM level.

Residue	ΔE^{int}	ΔE^{es}	ΔE^{ex}	ΔE^{ct+mix}	ΔE^{di}	ΔG_{sol}
Lys82	-141.385	-250.496	0.216	-0.555	-3.033	112.484
Leu197	-46.510	-12.336	0.271	-0.429	-2.375	-31.640
Gly196	-43.174	-43.062	0.018	-0.182	-0.735	0.788
Gly62	-34.317	-28.590	0.312	-0.380	-1.764	-3.896
Arg136	-26.851	-91.29	0	0	-0.895	65.333
Arg134	-17.438	-52.034	0.008	-0.001	-0.935	35.524
Ala65	-14.211	-21.054	-0.006	-0.008	-0.945	7.803
Cys133	-10.425	-5.720	-0.370	-0.148	-2.375	-1.813
Leu59	-9.960	-5.822	-0.060	-0.012	-1.960	-2.106
Val114	-7.320	-7.105	-0.016	0	-0.977	0.779
Phe183	-6.702	-1.999	-0.084	0.016	-5.790	1.155
Cys67	-6.390	-7.198	-0.182	-0.045	-3.571	4.606
Ala80	-6.080	-7.014	0.038	-0.003	-2.004	2.903
Leu130	-4.936	-4.423	-0.002	0	-1.087	0.576

Table S2. PIEDA of BI2536 and PLK1 complex (PDB ID: 2RKU). All energies are in kcal/mol. The calculation was conducted at the FMO-MP2/6-31G**/PCM level.

Residue	ΔE^{int}	ΔE^{es}	ΔE^{ex}	ΔE^{ct+mix}	ΔE^{di}	ΔG_{sol}
Cys133	-14.382	-15.497	12.292	-3.133	-7.068	-0.976
Gly60	-14.095	-13.257	5.720	-2.618	-5.916	1.976
Leu132	-12.557	-7.003	4.455	-2.848	-6.951	-0.21
Arg136	-11.995	-7.929	4.386	-2.637	-9.238	3.423
Leu59	-8.796	-1.082	7.484	-2.854	-12.121	-0.223
Phe183	-8.170	-1.082	3.851	-1.380	-8.944	-0.615
Arg57	-4.443	-4.717	1.939	-1.406	-3.641	3.382
Glu69	-4.255	-4.070	0.643	-0.980	-1.501	1.653
Cys67	-3.632	-3.406	7.750	-1.659	-6.159	-0.158
Gly62	-3.215	-2.520	1.025	-0.841	-2.058	1.179

Table S3. PIEDA of Onvansertib and PLK1 complex (PDB ID: 2YAC). All energies are in kcal/mol. The calculation was conducted at the FMO-MP2/6-31G**/PCM level.

Residue	ΔE^{int}	ΔE^{es}	ΔE^{ex}	ΔE^{ct+mix}	ΔE^{di}	ΔG_{sol}
Glu140	-49.684	-103.375	6.591	-5.569	-7.408	60.077
Lys82	-15.799	10.706	4.323	-2.501	-5.047	-23.280
Phe183	-12.410	-5.963	5.979	-2.432	-12.102	2.108
Arg134	-11.929	7.329	5.507	-2.883	-5.345	-16.537
Leu132	-10.461	-8.495	3.117	-2.183	-5.025	2.125
Cys133	-9.000	-5.721	8.138	-2.091	-6.214	-3.112
Gly60	-7.029	-12.922	2.082	-1.577	-3.544	8.932
Asn181	-6.527	-16.009	1.011	-0.727	-2.013	11.211
Leu59	-6.407	0.597	3.488	-1.644	-8.014	-0.834
Asp194	-5.387	-39.078	3.136	-2.553	-4.270	37.378
Arg136	-5.031	40.183	6.361	-3.800	-8.770	-39.005
Ser137	-4.245	-0.214	1.983	-0.862	-4.377	-0.775
Cys67	-3.444	-2.285	13.220	-3.305	-8.658	-2.416
Arg57	-3.129	23.939	0.892	-1.095	-1.710	-25.155

Table S4. PIEDA of GSK461364 and PLK1 complex. All energies are in kcal/mol. The calculation was conducted at the FMO-MP2/6-31G**/PCM level.

Residue	ΔE^{int}	ΔE^{es}	ΔE^{ex}	ΔE^{ct+mix}	ΔE^{di}	ΔG_{sol}
Glu140	-56.329	-120.777	13.802	-6.773	-8.460	65.879
Lys082	-12.529	0.356	10.821	-2.931	-6.761	-14.014
Phe183	-10.389	-8.297	6.331	-1.926	-10.167	3.670
Cys133	-10.248	-3.916	1.592	-0.251	-3.187	-4.486
Asp194	-9.998	-38.054	5.523	-3.781	-5.619	31.933
Asn181	-5.220	-12.858	0.545	-0.737	-2.068	9.898
Leu059	-4.066	0.449	2.725	-1.095	-5.254	-0.891
Gly060	-3.419	-12.033	0.704	-0.310	-2.527	10.747

Table S5. PIEDA of KBJK557 and PLK1 complex. All energies are in kcal/mol. The calculation was conducted at the FMO-MP2/6-31G**/PCM level.

Residue	ΔE^{int}	ΔE^{es}	ΔE^{ex}	ΔE^{ct+mix}	ΔE^{di}	ΔG_{sol}
His538	-25.291	-83.443	8.239	-4.695	-5.607	54.608
Lys540	-23.556	-94.849	3.000	-3.492	-4.191	71.785
Trp414	-21.232	-28.843	14.597	-5.024	-10.169	-1.962
Asp416	-10.779	14.983	6.886	-1.928	-4.024	-30.720
Tyr485	-8.698	-5.375	4.851	-1.775	-5.641	-6.399
Tyr417	-7.744	-2.724	4.970	-2.397	-8.491	-7.593
Leu490	-7.455	-2.355	6.972	-2.204	-9.725	-9.868
Val415	-6.996	-1.849	3.192	-2.662	-4.504	-5.677
Leu491	-5.033	-7.018	1.092	-0.442	-3.068	1.335
Phe535	-3.763	2.863	5.310	-2.895	-5.023	-9.041

Table S6. PIEDA of KBJK-4a and PLK1 complex. All energies are in kcal/mol. The calculation was conducted at the FMO-MP2/6-31G**/PCM level.

Residue	ΔE^{int}	ΔE^{es}	ΔE^{ex}	ΔE^{ct+mix}	ΔE^{di}	ΔG_{sol}
His538	-34.010	-110.056	21.855	-7.675	-6.445	68.311
Trp414	-16.658	-21.122	13.368	-4.167	-11.114	6.377
Lys540	-10.549	-59.446	2.091	-2.631	-2.826	52.263
Leu490	-7.450	-3.407	7.553	-2.552	-10.104	1.060
Phe535	-4.603	-0.517	1.522	-2.381	-3.560	0.333
Asp416	-4.029	19.404	5.679	-2.193	-3.998	-22.921
Thr539	-3.637	-4.309	0.006	0.143	-0.185	0.708
Val415	-3.564	2.558	1.164	-1.512	-3.257	-2.517
Tyr485	-3.540	-0.696	2.877	-0.921	-3.650	-1.150
Leu491	-3.347	-6.077	0.665	0.669	-2.375	3.771

Table S7. List of PDB IDs of protein structures used in this paper.

Entry	PDB ID	PLK1 domain	Ligands	Resolution (Å)
1	2OU7	KD	ATP	2.4
2	3FC2	KD	Volasertib	2.45
3	2RKU	KD	BI2536	1.95
4	4J52	KD	TAK-18	2.3
5	4J53	KD	TAK-960	2.5
6	5TA6	KD	Compound 15	2.5
7	5TA8	KD	Compound 11	2.6
8	2YAC	KD	Onvansertib	2.2
9	3KB7	KD	Compound 49	2.5
10	3THB	KD	MLN0905	2.5
11	4A4O	KD	Compound 13	2.7
12	4A4L	KD	Compound 25	2.35
13	3P37	PBD	FDPPLHSpTA	2.38
14	3RQ7	PBD	4j	1.55

KD: kinase domain

PBD: polo-box domain
